# Association Analysis of the *AGTR2:*rs1403543 Polymorphism with Newborn Kidney Size

**DOI:** 10.3390/genes17050554

**Published:** 2026-05-05

**Authors:** Karol Miler, Iwona Gorący, Beata Łoniewska, Klaudyna Lewandowska, Martyna Lica-Miler, Monika Rychel, Andrzej Ciechanowicz

**Affiliations:** 1 Department of Clinical and Molecular Biochemistry, Pomeranian Medical University, 70-111 Szczecin, Poland; kmiler01@gmail.com (K.M.); iwona.goracy@pum.edu.pl (I.G.); klaudyna.lewandowska@pum.edu.pl (K.L.); martyna.lica00@gmail.com (M.L.-M.);; 2 Neonatology and Neonatal Intensive Care Clinic, Pomeranian Medical University, 70-111 Szczecin, Poland; beata.loniewska@pum.edu.pl

**Keywords:** kidney size, genetic polymorphism, nephron endowment, newborns, type 2 receptor for angiotensin II

## Abstract

Background: The correlation between renal volume (or mass) and nephron number in newborns allows the use of the total kidney volume (TKV) at birth as a surrogate for congenital nephron number. Previously, the wide variation in final nephron number (termed “nephron endowment”) has been attributed to polymorphisms of genes encoding proteins involved in glomerulogenesis, including key genetic variants in the renin–angiotensin system. However, there are no data concerning the role of polymorphism in the gene encoding type-2 angiotensin II receptor (*AGTR2*) in the modulation of nephron endowment in humans. Therefore, the aim of our study was to analyze the possible association between *AGTR2*:rs1403543 polymorphism and kidney volume in Polish full-term healthy newborns. Methods: The study group consisted of 208 healthy, Polish, full-term newborns born to healthy women with uncomplicated pregnancies. The *AGTR2*:rs1403543 polymorphism was identified by PCR-RFLP in genomic DNA extracted from cord blood leukocytes. Kidney volume was measured sonographically. Total kidney volume was calculated as the sum of left and right kidneys, and normalized for body mass (BM), body length (BL), or body surface area (BSA). Results: There were no significant differences in TKV/BM, TKV/BL, or TKV/BSA between male and female newborns, as well as in regard to the *AGTR2*:rs1403543 polymorphism. Conclusions: The results suggest a lack of association between the *AGTR2*:rs1403543 polymorphism and physiological variability in kidney volume in full-term newborns.

## 1. Introduction

Brenner et al. hypothesized in 1988 that a reduced number of nephrons contributes to the development of primary hypertension in the general population [1]. The “proof of evidence” confirming Brenner’s hypothesis was provided in 2003 by Keller et al. [2]. Hinchliffe et al. stated that much of this variation in adult nephron number was likely due to variability in nephron endowment, i.e., the number of nephrons present at the end of nephrogenesis [3]. In humans, nephrogenesis begins at the 10th week and is completed by the 36th week of gestation (with 90% of nephrons developing during the third trimester), with a highly variable total number of nephrons, ranging from 200,000 to over 2 million [4,5,6,7]. Premature birth disrupts the development and maturation of the kidneys, leading to a reduction in the final number of nephrons [6]. Some researchers have also pointed to a link between low birth mass and a reduced number of nephrons in children [8]. Confirmation of a strong correlation between renal mass and nephron number in newborns allowed the establishment of sonographically measured kidney size/volume at birth (before the period of postnatal hypertrophy) as a surrogate for congenital nephron number [9]. Nephron endowment (congenital nephron number) is a multifactorial quantitative trait controlled by the interaction between fetomaternal environmental factors and numerous gene products that influence the extent of branching nephrogenesis during fetal life [10,11,12,13]. The branching of the ureteric bud (UB) is a crucial step during kidney organogenesis, and even minor developmental flaws during this process may markedly reduce nephron endowment [14].

Congenital anomalies of the kidney and urinary tract (CAKUT) occur at a frequency of 4–60 in 10,000 livebirths and are the most common cause of kidney failure in children worldwide [15,16,17,18]. The most common types of upper urinary tract CAKUT phenotypes include multicystic dysplastic kidneys, unilateral renal agenesis, and renal hypodysplasia, characterized by congenitally small kidneys with a reduced number of nephrons and dysplastic features [18,19].

The type-2 angiotensin II receptor in humans is encoded by the *AGTR2* gene located at chromosome Xq23. The common *AGTR2*:rs1403543G>A allele was revealed in 1999 by Nishimura et al. as a genetic variant associated with the predisposition to CAKUT in two independent cohorts of males: in American patients with ureteropelvic junction obstruction (UPJO) or multicystic dysplastic kidneys and German UPJO patients (both groups of European descent) [20]. However, further studies have reported inconsistent results [21,22,23,24,25]. Hiraoka et al. [21] in Japanese boys, and Siomou et al. [22] in children of European descent, reported comparable frequency distributions of the *AGTR2*:rs1403543 polymorphism between controls and CAKUT subjects as well as between the control group and CAKUT subgroups. In turn, Rigoli et al. [23] in Italian children, and Hahn et al. [24] in Korean children, reported a higher frequency of the *AGTR2*:rs1403543G allele in CAKUT patients as compared to control subjects. In 2014, Miranda et al., in Brazilian pediatric patients, did not confirm an association between rs1403543 or four other *AGTR2* polymorphisms (rs3736556, rs35474657, rs5193 and rs5194) with the predisposition to CAKUT in general [25]. However, the authors did indicate an association between rs3736556 or rs5194 polymorphisms (both variants in tight linkage disequilibrium with *AGTR2*:rs14003543) with the UPJO phenotype in these Brazilian subjects [25]. It is also worth noting that, due to the small total sample size of patients with renal maldevelopment, including renal hypodysplasia, studied so far [21,22,23,24,25], the association of *AGTR2*:rs14003543 with the risk of this CAKUT phenotype remains unclear.

Angiotensin II, as the key effector in the renin–angiotensin system, is an important stromal factor regulating UB branching [5]. Previously, we have found that the insertion/deletion polymorphism (rs4646994) in the gene encoding angiotensin I converting enzyme (rs4646994), as well as an interaction between genetic variants of angiotensinogen and type-1 angiotensin II receptor (rs5051 and rs5186), respectively, may account for the variation in kidney size at birth in full-term Polish newborns [10,26]. However, despite the confirmed role of signaling via stimulation of type-2 angiotensin II receptor in UB branching [27], there are no reports focused on the association of *AGTR2* polymorphism with the physiological variability of nephron number.

Therefore, taking into consideration the above data, we hypothesized that the common variants of the *AGTR2*:rs14003543 polymorphism might be associated with the physiological variability of congenital nephron number rather than with the risk of renal hypodysplasia.

To verify this hypothesis, we analyzed the possible association of *AGTR2*:rs14003543 variants with sonographically measured congenital kidney volume, a surrogate measure of the nephron endowment, in a cohort of Polish, full-term, healthy newborns without CAKUT phenotypes.

## 2. Materials and Methods

### 2.1. Newborns

The recruitment protocols, ultrasound examination techniques, and methodology for determining renal volume were conducted as previously reported [10]. The analyzed cohort included 208 full-term (gestational age > 37 weeks) healthy newborns of Polish ethnicity (96 females, 112 males). All participants were of European descent (descendants of people who arrived in Polish Western Pomerania after World War II from nearly all regions of the former Second Polish Republic), born to mothers who experienced physiological, non-pathological pregnancies. The study applied strict exclusion criteria, including multiple gestations, known chromosomal anomalies, and any congenital defects, with a specific focus on renal tract abnormalities identified via sonography. At the time of delivery, 500 μL of umbilical cord blood was collected for subsequent genomic DNA extraction. Clinical data, specifically newborn sex, birth mass (BM; kg), and body length (BL; m), were extracted from routine obstetric and neonatal records. To estimate the body surface area (BSA; m^2^), the Mosteller equation [28] was used: BSA = 0.0167 × (100 × BL)^0.5^ × BM^0.5^. On the third postnatal day, a single specialist performed the renal ultrasound examinations using an EnVisor C system (Philips Canada, Markham, ON, Canada). Depending on the clinical requirement, either a 5 MHz sector probe or a 10 MHz linear transducer was employed, following established protocols [29,30]. The volume for each kidney (left, LKV; right, RKV) was determined based on the prolate ellipsoid model [kidney volume = 4/3π (length/2) (height/2) (width/2)] [31]. Total kidney volume (TKV) was defined as the aggregate of LKV and RKV. To account for individual differences in neonatal growth, TKV was normalized to clinical anthropometric parameters, resulting in the following ratios: TKV per unit of body mass (TKV/BM), body length (TKV/BL), and body surface area (TKV/BSA). The research protocol adhered to the principles outlined in the latest Declaration of Helsinki (2024). Formal approval for the study was granted by the Bioethics Committee of the Pomeranian Medical University in Szczecin, Poland (decision No. BN-001/57/05). Prior to participation, written informed consent was secured from the parents or legal guardians of all enrolled newborns.

### 2.2. AGTR2:rs1403543 Genotyping

Isolation of genomic DNA from neonatal umbilical cord blood was performed using the QIAamp Blood DNA Mini Kit (QIAGEN, Dusseldorf, Germany) in strict accordance with the manufacturer’s instructions. To genotype the *AGTR2*:rs1403543 polymorphism, a PCR-RFLP (polymerase chain reaction-restriction fragment length polymorphism) approach was utilized. The amplification process employed the primers: 5′-GGAAAGTAGAACATACATTAAATG-3′ (forward) and 5′-CCTGTAAGAGAAACAGCAGCTAAAGAATT-3′ (reverse), synthesized by TIB MOL BIOL (Poznań, Poland). Following amplification, the *AGTR2* amplicons underwent digestion with the EcoRI restriction endonuclease (Thermo Fisher Scientific, Waltham, MA, USA): the G reference allele yielded two fragments (95 bp and 25 bp), whereas the A allele gave the intact 120 bp amplicon. These restriction products were subsequently resolved via electrophoresis on 3.5% agarose gels and visualized through Midori Green staining (Nippon Genetics Europe, Duren, Germany). To ensure high data reliability, 10% of the total samples (*n* = 21) were randomly selected for validation via Sanger sequencing, following previously established protocols [32]. The sequencing results showed 100% concordance with the PCR-RFLP findings. To eliminate bias, a double-blind genotyping procedure was implemented, where sample anonymization and laboratory analysis were conducted by separate investigators.

### 2.3. Statistical Analyses

Data normality was verified using Shapiro–Wilk tests. Due to non-normal distributions for most quantitative variables, results are presented as medians with minimum and maximum ranges. Group comparisons were performed via Mann–Whitney U or Kruskal–Wallis tests, while categorical data were assessed using chi-squared tests. To estimate the strength of an observed association, standardized effect sizes, such as Wilcoxon effect size (*r*) for Mann–Whitney U, *η**^2^*** for Kruskal–Wallis, and Cramér’s *V* for chi-squared tests were obtained. A *p*-value below 0.05 was set for statistical significance. A priori power analysis was conducted assuming small effect sizes (*r* = 0.1*, f* = 0.1, *V* = 0.1) for the available sample size; also, an analysis of the number of observations needed to achieve a test power of 80% was carried out. All computations were performed in R (version 4.5.2).

## 3. Results

In the female cohort, the *AGTR2*:rs1403543 genotype frequencies were 30.2% (*n* = 29) for GG homozygotes, 46.9% (*n* = 45) for GA heterozygotes, and 22.9% (*n* = 22) for AA homozygotes, with an A allele frequency of 46.3%. This distribution aligned with the Hardy–Weinberg equilibrium (*p* = 0.573). Among male newborns, 52.7% (*n* = 59) carried the G reference allele, while 47.3% (*n* = 53) possessed the A variant. Sex-specific clinical profiles and renal dimensions are detailed in Table 1. No significant sexual dimorphism was observed regarding body length, LKV, RKV, LKV/RKV ratio, or standardized kidney volumes (TKV/BM, TKV/BL, TKV/BSA). Conversely, males exhibited significantly higher values for BM, BSA, and TKV compared to females. All effects remained small (<0.3) with a priori power amounting to 0.108. To achieve a power of 0.8, more than 1571 observations would be required per group.

Analysis of *AGTR2*:rs1403543 variants revealed no significant associations with clinical parameters or kidney volumes in either females (Table 2) or males (Table 3). The analyzed polymorphism had a weak effect on variability among female anthropometric characteristics (*η*^2^ < 0.05) and a negligible effect (*η*^2^ = 0) on kidney volume, similarly in inheritance models and among males. The a priori power of Kruskal–Wallis ANOVA was 0.126, corresponding to a required sample size of 323 newborns per group to achieve a power of 0.8. The simulated predetermined power for Mann–Whitney U tests was 0.069 and 0.065 in female models, 0.082 among males. Each of these tests would require approximately 1571 observations per group to achieve a power of 0.8.

Further investigation using tertile (LT, MT or UT for lower, middle or upper tertile, respectively) distribution analysis for TKV indices confirmed a lack of significant correlation with *AGTR2*:rs1403543 variants in both sexes (Table 4 and Table 5). Association was negligible to small, with the power of tests ranging from 0.101 to 0.143. The required sample size needed to achieve a power of 0.8 ranged from 91 per group (LT vs. MT vs. UT; df = 4), up to 785 (LT vs. UT in female models or males; df = 1), or 964 (full inheritance models or LT vs. UT without models or males without models; df = 2).

To account for X-chromosome inactivation, GA heterozygous females were subsequently excluded. Following this exclusion, no significant inter-sex differences remained for any of the analyzed clinical or renal variables (Table 6). Observed effects were small, and the a priori computed power of the test was 0.092; the quantity necessary to obtain a power of 0.8 was similar to that of the other U tests and (1571 in each group).

Finally, no significant disparities in clinical characteristics or sonographic measurements were found when comparing G allele carriers (GG females and G hemizygous males) against A variant carriers (AA females and A hemizygous males) (Table 7), nor were any significant frequency shifts observed across TKV index tertiles (Table 8). No effects had a magnitude sufficient to be considered stronger than small. The simulated power of the Wilcoxon effect sizes for the independent two-sample test was equal to 0.096, and the power of the chi-squared test was computed as 0.190 (0.180 in LT vs. UT analysis). The counts needed to obtain adequate power remained unchanged.

## 4. Discussion

The present research investigated the potential link between the *AGTR2*:rs1403543 polymorphism and neonatal kidney dimensions, serving as a proxy for congenital nephron supply, in a population of healthy, full-term infants. Our analysis has suggested that *AGTR2* genetic variants do not significantly influence total kidney volume (TKV) when measured at 72 h post-delivery and normalized to body mass, length, or surface area. Given the X-chromosomal localization of the *AGTR2*:rs1403543 locus, sex-stratified analysis was initially performed to account for the genotypic differences between females (GG, GA, AA) and hemizygous males who carry either the rs1403543G or rs1403543A allele. Subsequently, a combined analysis involving both sexes was conducted to evaluate the broader impact of this polymorphism on TKV indices. However, to ensure an equal gene dosage, as performed similarly in a study by Alfakih et al. [33] and in our previous study [34], heterozygous female newborns were excluded from this subsequent analysis. An additional argument in favor of excluding female newborns heterozygous for the *AGTR2*:rs1403543 polymorphism was the possibility of the phenomenon of nonrandom (skewed) X-chromosome inactivation (XCI) in women leading to an allelic imbalance [35,36].

Liao et al. found that a deficiency in angiotensin II type 2 receptor in knockout (KO) mice diminished glomerulogenesis in Embryonic Day 15 embryos, but actual nephron numbers in KO neonates were only 2.5% smaller as compared to those in wild-type (WT) control mice [37]. It is also worth noting that the study by Liao et al. was carried out in only six KO neonates and five wild-type controls (n = 5), and the kidney mass to body mass ratio in knockout neonates was 6.7% lower than the value in control neonates [35]. It is also worth noting that angiotensin II type 2 receptor deficiency in knockout mice was associated with activation of ectopic hedgehog interacting protein (Hhip) gene expression, resulting either in podocyte loss or in transformation of differentiated podocytes to undifferentiated podocyte-derived fibrotic cells.

It has not escaped our notice that total kidney volume in our female newborns was significantly (5.7%) lower than that of male newborns. This may reflect the difference in nephron number consistently found in autopsy-based studies in adults [38,39,40]. In 1992, the analysis of 36 autopsy cases (18 females and 18 males) by Nyengaard et al. revealed that the mean glomerular number in females was about 10% lower than in males [38]. Highson et al. reported in 2006 that total glomerular number in female subjects was up to 15.6% lower than that of male subjects [39]. In turn, the study by McNamara et al., carried out in adult Senegalese Africans, revealed that females had 12.1% fewer glomeruli than males [40]. Conversely, no significant sexual dimorphism was observed regarding total kidney volumes in newborns once normalized for body mass, length, or surface area. Therefore, the above-mentioned results seem to reflect early sex-related anthropometric differences rather than real sex-related differences in nephron endowment. This suggests that glomerular number indexed to basic anthropometric parameters, rather than the absolute number of glomeruli, should be applied as the appropriate measurement in adults as a measure of nephron endowment.

Reports concerning the molecular effects of the *AGTR2*:rs1403543G>A transition are scarce [20,41]. Nishimura et al. demonstrated that the *AGTR2*:rs1403543 variant, situated in the lariat branchpoint motif of intron 1, interferes with mRNA splicing efficiency. Specifically, while the A allele-derived cDNA contains all three exons, the reference G allele cDNA is 60 nucleotides shorter because of exon 2 exclusion. Furthermore, G allele-derived mRNA expression was significantly reduced compared to the A allele levels [20]. Conversely, findings by Warnecke et al. suggested that G allele carriers might exhibit elevated levels of the type-2 angiotensin II receptor protein relative to A allele carriers [41]. One cannot rule out that while *AGTR2*:rs1403543 variants may directly impact transcription, this polymorphism could also be in strong linkage disequilibrium with other functionally critical *AGTR2* variants. It was suggested that *AGTR2* expression may be influenced partly by miR-208 a- and/or b-5p and that the C allele of the *AGTR2*:rs11091046 polymorphism (in tight linkage disequilibrium with the A allele of rs14003543) can reduce the binding affinity of miRNA to mRNA, leading to an increased amount of type-2 angiotensin II receptor protein [42]. It is also worth mentioning that *AGTR2*:rs1403543G>A is in tight linkage disequilibrium with the *AGTR2*:rs3736556 and rs5194 polymorphisms revealed previously by Miranda et al. as genetic variants associated with ureteropelvic junction obstruction, one of the major CAKUT phenotypes [25].

We are fully aware that a relatively small sample size (96 female newborns and 112 male newborns), resulting in a decrease in statistical power, represents a primary limitation of this research. This restricted cohort size stems from the fact that recruitment was finalized several years prior, and only in these 208 newborns did we obtain both the results of ultrasound measurements of kidney dimensions and good-quality genomic DNA samples. However, it should be emphasized that our study population is significantly larger than the total number of children analyzed so far (n = 156) for the association of *AGTR2*:rs1403543 polymorphism with the risk of renal maldevelopment (i.e., renal agenesis, renal aplasia, renal dysplasia and renal hypodysplasia) [21,22,23,24,25]. The frequency of the reference *AGTR2*:rs1403543G allele in the present study (53.7% or 52.7% in females or males, respectively) was similar to that reported in Brazilian healthy controls (56.4%) by Miranda et al. [25] but also much higher than in 102 healthy Japanese males (30.0%) analyzed by Hiraoka et al. [21] or in 100 healthy Koreans consisting the control group in the study by Hahn et al. [24].

Sonographically measured kidney volume is widely used as a surrogate for nephron numbers in newborns [8,10,11,26]. However, there are some doubts as to whether kidney volume can be fully reliable as a surrogate for nephron endowment [43]: First, measures of kidney volume are often not limited to parenchyma but also include such structures as renal pelvis or sinus fat [44]. Second, there is an inconsistency in the replication of sonographic measurements of kidney size. However, the intra-observer measurement errors are lower than inter-observer errors [45]. Therefore, sonographic measurements of all the newborns in our studied group were carried out by a single specialist using the same equipment. There are also other potential confounders (e.g., maternal factors, intrauterine environment and subtle perinatal variables) for the association analysis of *AGTR2*:rs1403543 polymorphism with newborn kidney volume. To reduce the impact of these, we have defined very strict exclusion and inclusion criteria for our study. Only healthy newborns born after the 37th week of gestation to healthy women with uncomplicated pregnancies (i.a., no diabetes in pregnancy, no gestational diabetes, no pregnancy-induced hypertension and no medication). In addition, the prevalence of smoking, arterial hypertension or obesity among mothers of newborns included in our study was very low.

## 5. Conclusions

Our results suggest a lack of association between the *AGTR2*:rs1403543 polymorphism and physiological variability of kidney volume in full-term newborns.

## Figures and Tables

**Table 1:** Clinical characteristics and kidney volumes of the newborns with regard to sex.

Variable	All Newborns (*n* = 208)	Female Newborns (*n* = 96)	Male Newborns (*n* = 112)	*p*	Effect Sizes (*r*) [95% CI]
BM [kg]	3.40 (2.36–5.09)	3.30 (2.36–4.30)	3.54 (2.36–5.09)	0.003	0.204 [0.060–0.350]
BL [m]	0.56 (0.44–0.63)	0.55 (0.46–0.63)	0.56 (0.44–0.63)	0.114	0.110 [0.007–0.240]
BSA [m^2^]	0.230 (0.170–0.289)	0.227 (0.174–0.270)	0.236 (0.170–0.289)	0.004	0.199 [0.070–0.340]
LKV [cm^3^]	12.2 (5.1–26.4)	11.8 (5.9–26.4)	12.6 (5.1–23.5)	0.088	0.118 [0.010–0.250]
RKV [cm^3^]	11.6 (5.4–26.9)	11.0 (6.1–26.9)	11.7 (5.4–25.3)	0.050	0.136 [0.020–0.270]
LKV/RKV	0.9 (0.4–2.0)	1.0 (0.6–1.6)	0.9 (0.4–2.0)	0.868	0.012 [0.003–0.160
TKV [cm^3^]	24.0 (10.6–53.3)	23.2 (12.0–53.3)	24.6 (10.6–46.2)	0.029	0.151 [0.020–0.290]
TKV/BM [cm^3^/kg]	7.1 (3.8–14.5)	7.0 (3.8–14.5)	7.2 (3.9–14.5)	0.544	0.128 [0.010–0.025]
TKV/BL [cm^3^/m]	43.0 (21.7–91.8)	41.6 (21.7–91.8)	44.3 (24.1–84.0)	0.065	0.042 [0.003–0.180]
TKV/BSA [cm^3^/m^2^]	104.5 (54.5–219.3)	102.8 (54.5–219.3)	106.4 (59.7–209.2)	0.201	0.089 [0.005–0.220]

Legend: Variables are presented as median, minimum and maximum. BM = Body Mass; BL = Body Length; BSA = Body Surface Area; LKV = Left Kidney Volume; RKV = Right Kidney Volume; TKV = Total Kidney Volume. The *p*-values are from Mann–Whitney tests. Effect sizes are Wilcoxon effect sizes (r) [CI = confidence interval].

**Table 2:** Clinical characteristics and kidney volumes of the female newborns in regard to *AGTR2*:rs1403543G>A.

Variable	GG (*n* = 29)	GA (*n* = 45)	AA (*n* = 22)	*p* _K-W_	Effect Sizes *(η^2^)* [95% CI]	*p* _D_	*p* _R_
BM [kg]	3.27 (2.36–4.30)	3.30 (2.48–4.22)	3.51 (2.75–4.30)	0.184	0.015 [0.000–0.140]	0.465	0.066
BL [m]	0.56 (0.46–0.63)	0.55 (0.50–0.63)	0.56 (0.51–0.60)	0.341	0.002 [0.000–0.110]	0.751	0.224
BSA [m^2^]	0.226 (0.174–0.270)	0.223 (0.185–0.270)	0.231 (0.199–0.268)	0.138	0.021 [0.000–0.150]	0.817	0.054
LKV [cm^3^]	11.7 (5.9–22.7)	11.5 (5.9–26.4)	12.3 (7.4–18.9)	0.525	0.000 [0.000–0.100]	0.952	0.282
RKV [cm^3^]	10.4 (6.1–15.3)	11.0 (6.3–26.9)	11.7 (6.2–17.1)	0.734	0.000 [0.000–0.070]	0.434	0.797
LKV/RKV	0.9 (0.6–1.2)	1.0 (0.7–1.6)	0.9 (0.6–1.2)	0.227	0.010 [0.000–0.130]	0.264	0.415
TKV [cm^3^]	23.8 (12.0–38.1)	22.7 (13.1–53.3)	23.7 (13.5–36.0)	0.667	0.000 [0.000–0.080]	0.655	0.376
TKV/BM [cm^3^/kg]	7.2 (3.8–10.3)	7.1 (4.4–14.5)	6.7 (4.2–12.8)	0.806	0.000 [0.000–0.080]	0.468	0.586
TKV/BL [cm^3^/m]	41.0 (21.7–70.5)	41.6 (24.6–91.8)	42.6 (26.5–66.6)	0.747	0.000 [0.000–0.070]	0.892	0.641
TKV/BSA [cm^3^/m^2^]	100.1 (54.5–162.0)	104.1 (62.2–219.3)	104.7 (63.4–175.2)	0.888	0.000 [0.000–0.060]	0.720	0.865

Legend: Variables are presented as median, minimum and maximum. The p _K-W_ values from Kruskal–Wallis tests, and the p _D_ or p _R_. values from Mann–Whitney tests in dominant (AA + GA versus GG) or recessive (AA versus GG + GA) mode of inheritance for the non-reference *AGTR2*:rs1403543A allele, respectively. Effect sizes are from *η^2^* for Kruskal–Wallis tests. For abbreviations, see Table 1.

**Table 3:** Clinical characteristics and kidney volumes of the male newborns in regard to *AGTR2*:rs1403543G>A.

Variable	G (*n* = 59)	A (n = 53)	*p*	Effect Sizes *(r)* [95% CI]
BM [kg]	3.65 (2.36–5.09)	3.39 (2.53–4.48)	0.097	0.157 [0.010–0.340]
BL [m]	0.57 (0.44–0.63)	0.56 (0.47–0.61)	0.160	0.133 [0.009–0.310]
BSA [m^2^]	0.243 (0.170–0.289)	0.229 (0.193–0.269)	0.075	0.169 [0.010–0.360]
LKV [cm^3^]	12.7 (5.1–22.1)	12.4 (7.4–23.5)	0.977	0.003 [0.003–0.210]
RKV [cm^3^]	11.6 (5.4–23.1)	11.7 (6.2–25.3)	0.886	0.014 [0.003–0.220]
LKV/RKV	0.9 (0.6–1.7)	1.0 (0.4–2.0)	0.875	0.015 [0.003–0.220]
TKV [cm^3^]	24.4 (10.6–43.5)	24.7 (14.3–46.2)	0.944	0.007 [0.003–0.220]
TKV/BM [cm^3^/kg]	43.3 (24.1–74.9)	44.5 (25.1–84.0)	0.675	0.040 [0.003–0.230]
TKV/BL [cm^3^/m]	7.2 (4.1–12.1)	7.3 (3.9–14.5)	0.506	0.063 [0.003–0.240]
TKV/BSA [cm^3^/m^2^]	106.3 (62.4–173.2)	106.6 (59.7–209.2)	0.624	0.047 [0.003–0.230]

Legend: Variables are presented as median, minimum and maximum. The *p*-values are from Mann–Whitney tests. Effect sizes are Wilcoxon effect sizes (r). For abbreviations, see Table 1.

**Table 4:** Frequency distributions of *AGTR2*:rs1403543 genotypes in female newborns in regard to tertiles of TKV indices.

Variable	Tertile	GG *n* (%)	GA *n* (%)	AA *n* (%)	*p*	Effect Sizes *V*	*p* _D_	Effect Sizes *V*	*p* _R_	Effect Sizes *V*
					LT vs. MT vs. UT (LT vs. UT)		LT vs. MT vs. UT (LT vs. UT)		LT vs. MT vs. UT (LT vs. UT)	
	Lower (<6.4)	11 (34)	15 (47)	6 (19)	0.723 (0.961)	0.104 (0.035)	0.708 (0.790)	0.085 (0.033)	0.389 (1.000)	0.140 (0.000)
TKV/BM [cm^3^/kg]	Middle (6.4–7.6)	8 (25)	14 (44)	10 (31)						
	Upper (>7.6)	10 (31)	16 (50)	6 (19)						
	Lower (<39.1)	11 (34)	15 (47)	6 (19)	0.891 (0.585)	0.076 (0.130)	0.708 (0.412)	0.085 (0.103)	0.662 (0.376)	0.093 (0.112)
TKV/BL [cm^3^/m]	Middle (39.1–45.8)	10 (31)	15 (47)	7 (22)						
	Upper (>45.8)	8 (25)	15 (47)	9 (28)						
	Lower (<92.6)	12 (38)	14 (44)	6 (19)	0.575 (0.854)	0.123 (0.070)	0.391 (0.599)	0.140 (0.066)	0.389 (1.000)	0.140 (0.000)
TKV/BSA [cm^3^/m^2^]	Middle (92.6–112.3)	7 (22)	15 (47)	10 (31)						
	Upper (>112.3)	10 (31)	16 (50)	6 (19)						

Legend: For abbreviations, see Table 1. The p, p _D_ or p _R_ values are from chi-squared tests for comparisons of GG versus GA versus AA and for comparisons in dominant or recessive mode of inheritance for the non-reference *AGTR2*:rs1403543A allele (AA + GA versus GG or AA versus GA + GG, respectively). LT versus MT versus UT or LT versus UT are for comparisons of lower tertile versus middle tertile versus upper tertile or for comparisons of lower tertile versus upper tertile, respectively. Effect sizes are Cramér’s *V* effect sizes.

**Table 5:** Frequency distributions of *AGTR2*:rs1403543 variants in hemizygous male newborns in regard to tertiles of TKV indices.

Variable	Tertile	G *n* (%)	A *n* (%)	*p* _LT vs. MT vs. UT_	Effect Sizes V	*p* _LT vs. UT_	Effect Sizes *V*
	Lower (<6.4)	19 (51)	18 (49)	0.784	0.066	0.816	0.081
TKV/BM [cm^3^/kg]	Middle (6.4–7.9)	22 (58)	16 (42)				
	Upper (>7.9)	18 (49)	19 (51)				
	Lower (<39.9)	21 (57)	16 (43)	0.711	0.078	0.816	0.027
TKV/BL [cm^3^/m]	Middle (39.9–48.2)	20 (53)	18 (47)				
	Upper (>48.2)	18 (49)	19 (51)				
	Lower (<97.7)	20 (54)	17 (46)	0.831	0.058	0.642	0.054
TKV/BSA [cm^3^/m^2^]	Middle (97.7–116.6)	21 (55)	17 (45)				
	Upper (>116.6)	18 (49)	19 (51)				

Legend: For abbreviations, see Table 1. The *p*-values are from chi-squared tests for comparisons of lower tertile versus middle tertile versus upper tertile (LT vs. MT vs. UT) or lower tertile versus upper tertile (LT vs. UT), respectively. Effect sizes are Cramér’s *V* effect sizes.

**Table 6:** Clinical characteristics and kidney volumes of the newborns with regard to sex after exclusion of GA *AGTR2* heterozygous females.

Variable	Female Newborns (*n* = 29 + 22)	Male Newborns (*n* = 112)	*p*	Effect Sizes (*r*) [95% CI]
BM [kg]	3.32 (2.36–4.30)	3.54 (2.36–5.09)	0.054	0.051 [0.020–0.300]
BL [m]	0.56 (0.46–0.63)	0.56 (0.44–0.63)	0.554	0.046 [0.002–0.200]
BSA [m^2^]	0.229 (0.174–0.270)	0.236 (0.170–0.289)	0.110	0.125 [0.008–0.280]
LKV [cm^3^]	11.8 (5.9–22.7)	12.6 (5.1–23.5)	0.284	0.084 [0.004–0.220]
RKV [cm^3^]	10.8 (6.1–17.1)	11.7 (5.4–25.3)	0.051	0.153 [0.020–0.290]
LKV/RKV	0.9 (0.6–1.2)	0.9 (0.4–2.0)	0.398	0.066 [0.003–0.220]
TKV [cm^3^]	23.8 (12.0–38.1)	24.6 (10.6–46.2)	0.080	0.137 [0.010–0.270]
TKV/BM [cm^3^/kg]	6.7 (3.8–12.8)	7.2 (3.9–14.5)	0.090	0.133 [0.009–0.270]
TKV/BL [cm^3^/m]	41.7 (21.7–70.5)	44.3 (24.1–84.0)	0.418	0.064 [0.003–0.210]
TKV/BSA [cm^3^/m^2^]	101.1 (54.5–175.2)	106.4 (59.7–209.2)	0.173	0.107 [0.005–0.250]

Legend: Variables are presented as median, minimum and maximum. The *p*-values are from Mann–Whitney tests. Effect sizes are Wilcoxon effect sizes (*r*). For abbreviations, see Table 1.

**Table 7:** Clinical characteristics and kidney volumes of the newborns in regard to *AGTR2*:rs1403543G>A after exclusion of GA heterozygous females.

Variable	GG + G (*n* = 29 + 59)	AA + A (*n* = 22 + 53)	*p*	Effect Sizes (*r*) [95% CI]
BM [kg]	3.46 (2.36–5.09)	3.46 (2.53–4.48)	0.552	0.047 [0.003–0.190]
BL [m]	0.56 (0.44–0.63)	0.56 (0.47–0.61)	0.467	0.060 [0.003–0.210]
BSA [m^2^]	0.232 (0.170–0.289)	0.230 (0.193–0.269)	0.389	0.068 [0.004–0.210]
LKV [cm^3^]	12.2 (5.1–22.7)	12.4 (7.4–23.5)	0.764	0.024 [0.004–0.180]
RKV [cm^3^]	11.5 (5.4–23.1)	11.7 (6.2–25.3)	0.809	0.019 [0.002–0.180]
LKV/RKV	0.9 (0.6–1.7)	0.9 (0.4–2.0)	0.887	0.011 [0.002–0.180]
TKV [cm^3^]	24.0 (10.6–43.5)	24.7 (13.5–46.2)	0.591	0.042 [0.003–0.190]
TKV/BM [cm^3^/kg]	7.2 (3.8–12.1)	7.1 (3.9–14.5)	0.710	0.064 [0.005–0.220]
TKV/BL [cm^3^/m]	42.8 (21.7–74.9)	43.8 (25.1–84.0)	0.418	0.029 [0.003–0.190]
TKV/BSA [cm^3^/m^2^]	104.5 (54.5–173.2)	105.1 (59.7–209.2)	0.621	0.039 [0.003–0.190]

Legend: Variables are presented as median, minimum and maximum. The *p*-values are from Mann–Whitney tests. Effect sizes are Wilcoxon effect sizes (*r*). For abbreviations, see Table 1.

**Table 8:** Frequency distributions of *AGTR2*:rs1403543 variants in GG homozygous females and G hemizygous males and in AA homozygous females and A hemizygous males in regard to tertiles of total kidney volumes.

Variable	Tertile	GG + G *n* (%)	AA + A *n* (%)	*p* _LT vs. MT vs. UT_	Effect Sizes *V*	*p* _LT vs. UT_	Effect Sizes *V*
	Lower (<6.4)	31 (57)	23 (43)	0.658	0.072	0.846	0.019
TKV/BM [cm^3^/kg]	Middle (6.4–7.8)	27 (49)	28 (51)				
	Upper (>7.8)	30 (56)	24 (44)				
	Lower (<39.9)	31 (57)	23 (43)	0.738	0.061	0.440	0.074
TKV/BL [cm^3^/m]	Middle (39.9–47.3)	30 (55)	25 (45)				
	Upper (>47.3)	27 (50)	27 (50)				
	Lower (<96.0)	31 (57)	23 (43)	0.823	0.049	0.562	0.056
TKV/BSA [cm^3^/m^2^]	Middle (96.0–114.9)	29 (53)	26 (47)				
	Upper (>114.9)	28 (52)	26 (48)				

Legend: For abbreviations, see Table 1. The *p*-values are from chi-squared tests for comparisons of lower tertile versus middle tertile versus upper tertile (LT vs. MT vs. UT) or lower tertile versus upper tertile (LT vs. UT), respectively. Effect sizes are Cramér’s *V* effect sizes. For abbreviations, see Table 1.

## Data Availability

The data are available from the corresponding author upon reasonable request.

## Abbreviations

The following abbreviations are used in this manuscript:

TKV	Total Kidney Volume
*AGTR2*	Type-2 Angiotensin II Receptor gene
PCR RFLP	Polymerase Chain Reaction Restriction Fragment Length Polymorphism
DNA	Deoxyribonucleic Acid
BM	Body Mass
BL	Body Length
BSA	Body Surface Area
UB	Ureteric Bud
CAKUT	Congentital Anomalies of the Kidney and Urinary Tract
UPJO	Ureteropelvic Junction Obstruction
LKV	Left Kidney Volume
RKV	Right Kidney Volume
LT	Lower Tertile
MT	Middle Tertile
UT	Upper Tertile
CI	Confidence Interval
XCI	X-Chromosome Inactivation
KO	Knockout
WT	Wild Type
mRNA	messenger Ribonucleic Acid
cDNA	complementary Deoxyribonucleic Acid

## References

[1] Brenner B.M., Garcia D.L., Anderson S. (1988). Glomeruli and blood pressure. Less of one, more the other?. Am. J. Hypertens..

[2] Keller G., Zimmer G., Mall G., Ritz E., Amann K. (2003). Nephron number in patients with primary hypertension. N. Engl. J. Med..

[3] Hinchliffe S.A., Sargent P.H., Howard C.V., Chan Y.F., van Velzen D. (1991). Human intrauterine renal growth expressed in absolute number of glomeruli assessed by the disector method and Cavalieri principle. Lab. Investig..

[4] Imberti B., Benigni A. (2025). Renal Endowment in Men and Women: Start from the Beginning. Nephron.

[5] Yosypiv I.V. (2014). Renin-angiotensin system in ureteric bud branching morphogenesis: Implications for kidney disease. Pediatr. Nephrol..

[6] Villani G., Zaza P., Lamparelli R., Maffei G. (2024). Kidney volume-to-birth weight ratio as an estimate of nephron endowment in extremely low birth weight preterm infants. Sci. Rep..

[7] Chevalier R.L. (2023). CAKUT: A Pediatric and Evolutionary Perspective on the Leading Cause of CKD in Childhood. Pediatr. Rep..

[8] Luyckx V.A., Brenner B.M. (2020). Clinical consequences of developmental programming of low nephron number. Anat. Rec..

[9] Zhang Z., Quinlan J., Hoy W., Hughson M.D., Lemire M., Hudson T., Hueber P.A., Benjamin A., Roy A., Pascuet E. (2008). A common RET variant is associated with reduced newborn kidney size and function. J. Am. Soc. Nephrol..

[10] Kaczmarczyk M., Łoniewska B., Kuprjanowicz A., Józwa A., Bińczak-Kuleta A., Gorący I., Dawid G., Kordek A., Karpińska-Kaczmarczyk K., Brodkiewicz A. (2013). An insertion/deletion ACE polymorphism and kidney size in Polish full-term newborns. J. Renin-Angiotensin-Aldosterone Syst..

[11] El Kares R., Manolescu D.C., Lakhal-Chaieb L., Montpetit A., Zhang Z., Bhat P.V., Goodyer P. (2010). A human ALDH1A2 gene variant is associated with increased newborn kidney size and serum retinoic acid. Kidney Int..

[12] Moritz K.M., Wintour E.M., Black M.J., Bertram J.F., Caruana G. (2008). Factors Influencing Mammalian Kidney Development: Implications for Health in Adult Life.

[13] Hoy W.E., Ingelfinger J.R., Hallan S., Hughson M.D., Mott S.A., Bertram J.F. (2010). The early development of the kidney and implications for future health. J. Dev. Orig. Health Dis..

[14] Sakurai H., Nigam S.K. (1998). In vitro branching tubulogenesis: Implications for developmental and cystic disorders, nephron number, renal repair, and nephron engineering. Kidney Int..

[15] Matsell D.G., Catapang M., Becknell B. (2023). Predicting outcomes in children with congenital anomalies of the kidney and urinary tract. Pediatr. Nephrol..

[16] Zwolińska D., Polak-Jonkisz D., Makulska I. (2011). Podłoże genetyczne wrodzonych wad nerek i układu moczowego [Genetics of congenital anomalies of the kidney and urinary tract]. Postep. Hig. I Med. Dosw..

[17] Koenigbauer J.T., Fangmann L., Reinhardt C., Weichert A., Henrich W., Saskia B., Gabriel H.P. (2024). Spectrum of congenital anomalies of the kidney and urinary tract (CAKUT) including renal parenchymal malformations during fetal life and the implementation of prenatal exome sequencing (WES). Arch. Gynecol. Obstet..

[18] Walawender L., Becknell B., Matsell D.G. (2023). Congenital anomalies of the kidney and urinary tract: Defining risk factors of disease progression and determinants of outcomes. Pediatr. Nephrol..

[19] Katsoufis C.P. (2020). Clinical predictors of chronic kidney disease in congenital lower urinary tract obstruction. Pediatr. Nephrol..

[20] Nishimura H., Yerkes E., Hohenfellner K., Miyazaki Y., Ma J., Hunley T.E., Yoshida H., Ichiki T., Threadgill D., Phillips J.A. (1999). Role of the angiotensin type 2 receptor gene in congenital anomalies of the kidney and urinary tract, CAKUT, of mice and men. Mol. Cell..

[21] Hiraoka M., Taniguchi T., Nakai H., Kino M., Okada Y., Tanizawa A., Tsukahara H., Ohshima Y., Muramatsu I., Mayumi M. (2001). No evidence for AT2R gene derangement in human urinary tract anomalies. Kidney Int..

[22] Siomou E., Bouba I., Kollios K.D., Papadopoulou F., Syrrou M., Georgiou I., Siamopoulou A. (2007). Angiotensin II type 2 receptor gene polymorphism in Caucasian children with a wide spectrum of congenital anomalies of the kidney and urinary tract. Pediatr. Res..

[23] Rigoli L., Chimenz R., di Bella C., Cavallaro E., Caruso R., Briuglia S., Fede C., Salpietro C.D. (2004). Angiotensin-converting enzyme and angiotensin type 2 receptor gene genotype distributions in Italian children with congenital uropathies. Pediatr. Res..

[24] Hahn H., Ku S.E., Kim K.S., Park Y.S., Yoon C.H., Cheong H.I. (2005). Implication of genetic variations in congenital obstructive nephropathy. Pediatr. Nephrol..

[25] Miranda D.M., Dos Santos A.C., Sarubi H.C., Bastos-Rodrigues L., Rosa D.V., Freitas I.S., De Marco L.A., Oliveira E.A., Simões e Silva A.C. (2014). Association of angiotensin type 2 receptor gene polymorphisms with ureteropelvic junction obstruction in Brazilian patients. Nephrology.

[26] Kaczmarczyk M., Kuprjanowicz A., Łoniewska B., Gorący I., Taryma-Leśniak O., Skonieczna-Żydecka K., Ciechanowicz A. (2015). Epistatic interaction between common *AGT* G(−6)A (rs5051) and *AGTR1* A1166C (rs5186) variants contributes to variation in kidney size at birth. Gene.

[27] Song R., Spera M., Garrett C., El-Dahr S.S., Yosypiv I.V. (2010). Angiotensin II AT2 receptor regulates ureteric bud morphogenesis. Am. J. Physiol. Renal. Physiol..

[28] Mosteller R.D. (1987). Simplified calculation of body-surface area. N. Engl. J. Med..

[29] Schmidt I.M., Main K.M., Damgaard I.N., Mau C., Haavisto A.M., Chellakooty M., Boisen K.A., Petersen J.H., Scheike T., Olgaard K. (2004). Kidney growth in 717 healthy children aged 0–18 months: A longitudinal cohort study. Pediatr. Nephrol..

[30] Vujic A., Kosutic J., Bogdanovic R., Prijic S., Milicic B., Igrutinovic Z. (2007). Sonographic assessment of normal kidney dimensions in the first year of life—A study of 992 healthy infants. Pediatr. Nephrol..

[31] Quinlan J., Lemire M., Hudson T., Qu H., Benjamin A., Roy A., Pascuet E., Goodyer M., Raju C., Zhang Z. (2007). A common variant of the PAX2 gene is associated with reduced newborn kidney size. J. Am. Soc. Nephrol..

[32] Dmytrzak A., Lewandowska K., Boroń A., Łoniewska B., Grzesch N., Brodkiewicz A., Clark J.S.C., Ciechanowicz A., Kostrzewa-Nowak D. (2024). No Association of Polymorphisms in the Genes Encoding Interleukin-6 and Interleukin-6 Receptor Subunit Alpha with the Risk of Keloids in Polish Patients. Int. J. Mol. Sci..

[33] Alfakih K., Maqbool A., Sivananthan M., Walters K., Bainbridge G., Ridgway J., Balmforth A.J., Hall A.S. (2004). Left ventricle mass index and the common, functional, X-linked angiotensin II type-2 receptor gene polymorphism (−1332 G/A) in patients with systemic hypertension. Hypertension.

[34] Gorący I., Miler K., Lewandowska K., Rychel M., Łoniewska B., Ciechanowicz A. (2025). The rs1403543 Polymorphism of *AGTR2*, Which Encodes the Type-2 Angiotensin II Receptor, and Left Ventricular Mass in Polish Full-Term Newborns. Genes.

[35] Fu Y., Tan X., Qin L., Wang C. (2025). From inactivation to intervention: X chromosome silencing in disease pathogenesis and emerging therapeutic strategies. Genes Dis..

[36] Minks J., Robinson W.P., Brown C.J. (2008). A skewed view of X chromosome inactivation. J. Clin. Investig..

[37] Liao M.C., Zhao X.P., Chang S.Y., Lo C.S., Chenier I., Takano T., Ingelfinger J.R., Zhang S.L. (2017). AT_2_R deficiency mediated podocyte loss via activation of ectopic hedgehog interacting protein (*Hhip*) gene expression. J. Pathol..

[38] Nyengaard J.R., Bendtsen T.F. (1992). Glomerular number and size in relation to age, kidney weight, and body surface in normal man. Anat. Rec..

[39] Hughson M.D., Douglas-Denton R., Bertram J.F., Hoy W.E. (2006). Hypertension, glomerular number, and birth weight in African Americans and white subjects in the southeastern United States. Kidney Int..

[40] McNamara B.J., Diouf B., Hughson M.D., Douglas-Denton R.N., Hoy W.E., Bertram J.F. (2008). Renal pathology, glomerular number and volume in a West African urban community. Nephrol. Dial. Transplant..

[41] Warnecke C., Mugrauer P., Sürder D., Erdmann J., Schubert C., Regitz-Zagrosek V. (2005). Intronic ANG II type 2 receptor gene polymorphism 1675 G/A modulates receptor protein expression but not mRNA splicing. Am. J. Physiol. Regul. Integr. Comp. Physiol..

[42] Yvert T.P., Zempo H., Gabdrakhmanova L.J., Kikuchi N., Miyamoto-Mikami E., Murakami H., Naito H., Cieszczyk P., Leznicka K., Kostryukova E.S. (2018). AGTR2 and sprint/power performance: A case-control replication study for rs11091046 polymorphism in two ethnicities. Biol. Sport.

[43] Luyckx V.A., Shukha K., Brenner B.M. (2011). Low nephron number and its clinical consequences. Rambam Maimonides Med. J..

[44] Denic A., Elsherbiny H., Rule A.D. (2019). In-vivo techniques for determining nephron number. Curr. Opin. Nephrol. Hypertens..

[45] Eze C.U., Eze C.U., Marchie T.T., Ohagwu C.C., Ochie K. (2013). Observer Variability in Sonographic Measurement of Kidney Sizes among Children in Benin-City, Nigeria. West Indian Med. J..

